# Synthesis and Characterization of TiO_2_ Nanoparticles for the Reduction of Water Pollutants

**DOI:** 10.3390/ma10101208

**Published:** 2017-10-20

**Authors:** Gigliola Lusvardi, Corrado Barani, Federica Giubertoni, Giulia Paganelli

**Affiliations:** 1 Department of Chemistry and Geological Sciences, University of Modena and Reggio Emilia, Via G. Campi 103, 41125 Modena, Italy; giulipez91@gmail.com; 2 Barchemicals, Via S. Allende 14, 41051 Castelnuovo Rangone (MO), Italy; barani.corrado@barchemicals.it (C.B.); giubertoni.federica@barchemicals.it (F.G.)

**Keywords:** TiO_2_, nanoparticles, photocatalysis, pollutants

## Abstract

The aim of this manuscript was the optimization of the synthesis of TiO_2_ nanoparticles (TiO_2_ NPs) with conditions that could be easily reproducible at the industrial level. Several procedures were tested and those with C_12_H_28_O_4_Ti and CO(NH_2_)_2_ as precursors seemed the most promising and, consequently, were improved with different molar ratios, lower temperatures and the addition of NH_4_Cl as a secondary dopant of nitrogen. The obtained samples were studied with analytical techniques such as X-ray powder diffraction (XRPD) and field emission scanning electron microscopy (FESEM). To complete the study, dye degradation and bacteriological tests were also performed. The results indicate that it is possible to obtain TiO_2_ NPs at lower temperatures with respect to those used in the literature; the best candidate that could satisfy all the requirements was a sample with a molar ratio of C_12_H_28_O_4_Ti:CO(NH_2_)_2_ at 2:1 and obtained at 50 °C.

## 1. Introduction

Nanotechnologies are a set of methods and techniques for the treatment of matter and aimed at obtaining materials with novel functionalities and improved characteristics. Among the various materials, nanoparticles play a special role in a wide range of applications and, in particular, there are a large number of studies related to titanium dioxide nanoparticles (TiO_2_ NPs) [1,2,3]. The TiO_2_ NPs studied and synthesized since the twentieth century have been involved in large-scale production, thanks to several uses (sunscreen, paints, toothpastes, and so on). Other uses concerned air purification, in particular the reduction of nitrogen oxides (NOx), sulfur oxides (SOx), carbon monoxide (CO), aromatic hydrocarbons BTX (benzene, toluene, xylene), and polycondensed aromatic hydrocarbons, atmospheric particulate PM_10_ (with high concentrations in urban areas), and volatile organic compounds (VOCs) released by paints and plasters in adjacent environments (indoor pollution) [4,5,6,7,8].

Numerous studies have reported the properties of titanium dioxide and its use for the degradation of substances in an aqueous solution and the reduction of inorganic ions [9,10], and TiO_2_ has been considered the most widely used oxide in photocatalysis. ZnO and α-Fe_2_O_3_ can be also used as photocatalysts; α-Fe_2_O_3_ absorbs visible light, but with lower photocatalytic activity than that of TiO_2_ and ZnO, which has the further disadvantage of releasing Zn^2+^ ions in aqueous solution [11]. Consequently, attention was devoted to TiO_2_, especially in the crystalline anatase phase [12,13], and was utilized for the preparation of self-cleaning surfaces that are in contact with pool water (or water in general) and for the coating of materials, such as ceramic and natural stone, with anti-algae properties [14,15,16,17,18,19,20,21]. Unfortunately, the band gap of anatase (3.2 eV) was not ideal for solar applications, with a consequent limitation for applications in the visible spectral range. It is well known that the positive effect of doping with different elements (transition metals or non-metallic elements) improves the photocatalytic properties of TiO_2_ [22,23,24,25]. 

It is common sense that the literature is very rich in titanium dioxide papers; for example, research conducted on a well-rounded search engine, with only the title “titanium dioxide” lead to our obtaining, for 2017 only, over 1,400,000 manuscripts, and refining the search to “nanoparticles”, “photocatalysis”, “synthesis”, and “temperature”, found that about 50 manuscripts were listed. The interest derives from a synthesis that can be easily achieved in the laboratory at low temperature, without heavy metals and dangerous reagents; this kind of approach, in our opinion, does not emerge from the literature. We focused on some manuscripts from 2017 [26,27,28], which while being close to our needs do not fully meet our requirements.

The study reported in this manuscript comes from research developed in collaboration with the company that is among the co-authors of this work. This company has operated since 1984 in the water treatment and swimming pool chemical products sector for the conditioning and treatment of all types of water. The company’s need for was to find a synthesis that can be easily reproduced in a laboratory at the industrial scale. For this reason, we focused our attention on some of the syntheses of TiO_2_ NPs mentioned in the literature [29,30,31,32,33,34,35,36,37,38], generally deriving from a hydrothermal process and/or sol-gel route.

The purpose of the present manuscript is the determination of an improved synthesis of TiO_2_ NPs with conditions that could be easily reproducible in industry, both in terms of energy saving and cost reduction. These materials will be characterized by means of analytical techniques, such as X-ray powder diffraction (XRPD) and field emission scanning electron microscopy (FESEM); dye degradation and bacteriological tests will be performed successively. These last tests are very important in order to verify the effectiveness in the removal of bacterial agents of specific colonies.

## 2. Methods

### 2.1. Synthesis

Some selected procedures reported in the literature were tested and reproduced in our laboratory; subsequently, they were optimized and improved in order to obtain TiO_2_ NPs in the best, most simple and inexpensive procedure, in agreement with the market requirements.

The most commonly used reagents were titanium tetrachloride (TiCl_4_), 99%; titanium isopropoxide (C_12_H_28_O_4_Ti), ≥99.8%; urea (CO(NH_2_)_2_), 99%; ammonium chloride (NH_4_Cl), 99.5%; glacial acetic acid, 99.5%; methanol, 99.5%; and ethanol 99.8%.

In the subsequent Table 1, Table 2 and Table 3, we report three tested syntheses and a short summary of the related procedures. 

Ethanol and titanium tetrachloride were introduced into a beaker; the solution was stirred for 30 min. During this period, it formed a yellow sol phase. Bidistilled water was added and the solution became clear and colorless. The solution was again stirred for 30 min at room temperature and then the formed gel was dried at 50 °C for 24 h.

Bidistilled water and urea were introduced into a beaker and the solution was stirred for five minutes. Titanium isopropoxide was added dropwise and the obtained suspension was stirred for 30 min. This suspension was introduced to a water bath for one hour at 90 °C; the separated product was dried at 80 °C for 12 h.

Isopropyl alcohol and titanium isopropoxide were introduced into a beaker and the solution was stirred for one hour; after this acetic acid and methanol were added. The solution was dried at 90 °C for 24 h until a yellow powder was obtained. 

The diffractometric analysis (discussed later) performed for these samples provided the possibility to verify the formation of anatase and to evaluate the ability of the nitrogen dopant to enhance the photocatalytic properties. 

The second synthesis was selected and improved. This synthesis was optimized by means of the use of different molar ratios between C_12_H_28_O_4_Ti and CO(NH_2_)_2_, different temperatures, and the addition of another dopant containing nitrogen (NH_4_Cl). NH_4_Cl was dissolved in an aqueous solution of CO(NH_2_)_2_ and the process was the same as described for Synthesis 2. 

These new parameters (Synthesis 4) gave rise to a set of samples reported in Table 4.

All samples were tested at 50 °C and room temperature (r.t.), except for the samples with NH_4_Cl, where the used temperature was always 50 °C.

### 2.2. Dye Degradation Tests

These tests are qualitative and were used to verify the possibility to degrade a dye. The dyes used were methyl orange and bromothymol blue [39]. The tests were carried out only on selected samples: samples with molar ratios C_12_H_28_O_4_Ti:CO(NH_2_)_2_ 10:1 and 2:1 obtained at 50 °C and at room temperature. Two grams of the samples were added to 50 mL of a solution (20%) of isopropyl alcohol and the obtained suspension was divided into two beakers containing, respectively, 0.25 mL of methyl orange and 0.25 mL of bromothymol blue. The concentration of the dyes was approximately 6 × 10^−3^ M. The samples were exposed to sunlight for a few hours.

Another test was also performed for samples with the molar ratios C_12_H_28_O_4_Ti:CO(NH_2_):NH_4_Cl 10:1:0, 2:1:0, 10:1:0.52, and 2:1:0.52 obtained at 50 °C. The samples were prepared as previously described and the obtained suspension was deposited on ceramic tiles which were exposed to sunlight for a few hours. The use of ceramic tiles was justified by the need to simulate coatings in contact with water, for example in a swimming pool.

### 2.3. Bacteriological Tests

These tests were performed on ceramic tiles with samples with molar ratios of C_12_H_28_O_4_Ti:CO(NH_2_):NH_4_Cl 2:1:0, 2:1:0.52 at 50 °C. The tiles were introduced to contaminated water and exposed to sunlight. After predefined times (5, 30, 120, and 240 min) the solution was analyzed in order to monitor the number of bacterial strains [40].

### 2.4. Characterization

A mineralogical analysis was performed by X-ray powder diffraction (XRPD) measurements that were carried out for all the specimens in the 5–60°, 2θ range, employing an X’Pert PANAnalytical apparatus (PANalytical, Almeno, The Netherlands), equipped with Ni-filtered Cu Kα radiation (λ = 1.54060 Å). The identification of the crystalline phases was based on the Joint Committee on Powder Diffraction Standards (JCPDS) provided from International Centre for Diffraction Data database [41]. The crystallite size of the nanoparticles was calculated using Scherrer’s equation [42]. Morphological investigation and evaluation of the dimensions of the nanoparticles was performed by a Nova NanoSEM 450 field emission instrument (FEI CORPORATE HEADQUARTERS, Hillsboro, OR, USA).

## 3. Results and Discussion

The tested and related characterization results of all syntheses are reported below.

### 3.1. Diffractometric Analysis

The diffractograms allowed us to recognize the crystalline forms of TiO_2_ for the different syntheses; the original powder patterns are reported as Supplementary Materials (Figures S1–S9) and are characteristic of nanometric powders. The studied 2θ range was 5–60°, but for better interpretation we report the 2θ range between 10–50°.

We report in Table 5 and Table 6 the comparison of the most important values from the theoretical and experimental d (interplanar distance), 2θ (angles), and I% (relative intensity) for TiO_2_ (anatase).

Only for Synthesis 1 and 2 was it possible to recognize TiO_2_ in the anatase form. In particular for Synthesis 2, the peaks were more intense and best solved; consequently, this synthesis was improved (as already explained) and the results are reported in Table 6.

In all cases it was possible recognize TiO_2_ in the anatase form; it is also important to note that the synthesis was carried out at lower temperatures or with another dopant allowing us to obtain, again, TiO_2_ in the anatase form.

### 3.2. Morphological Analysis

In general it was possible observe that the TiO_2_NPs in term of distribution, shape, and dimensions were quite regular, because sometimes the nanoparticles were aggregates. We summarize the results in term of shape and distribution in Table 7.

**Figure 1:**
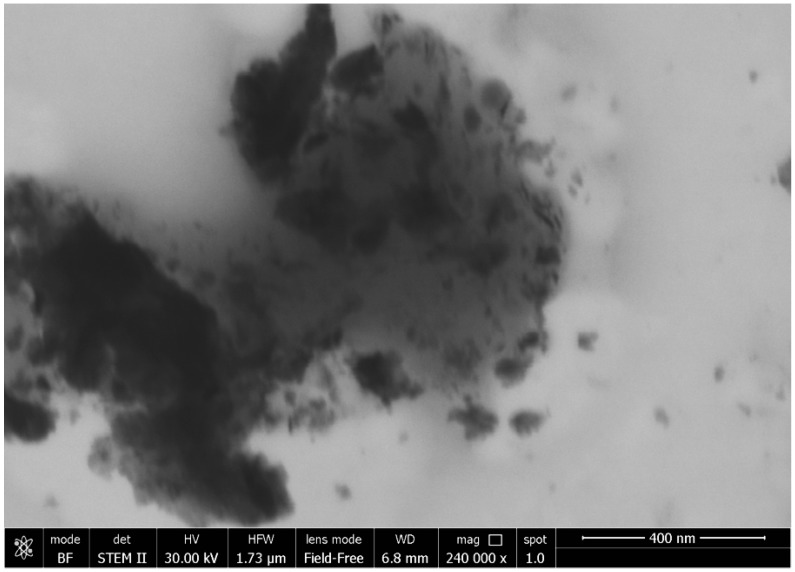
Field Emission Scanning Electron Micrograph (FESEM) of TiO_2_ NPs with C_12_H_28_O_4_Ti:CO(NH_2_)_2_ at a ratio of 2:1, at 50 °C.

The best conditions of synthesis (as reported in Table 7) were those with C_12_H_28_O_4_Ti:CO(NH_2_)_2_ in a 2:1 ratio, and obtained at 50 °C (Figure 1); the darker nanoparticles are constituted of TiO_2_ NPs and the clearest surrounding area is constituted of the organic matrix.

**Figure 2:**
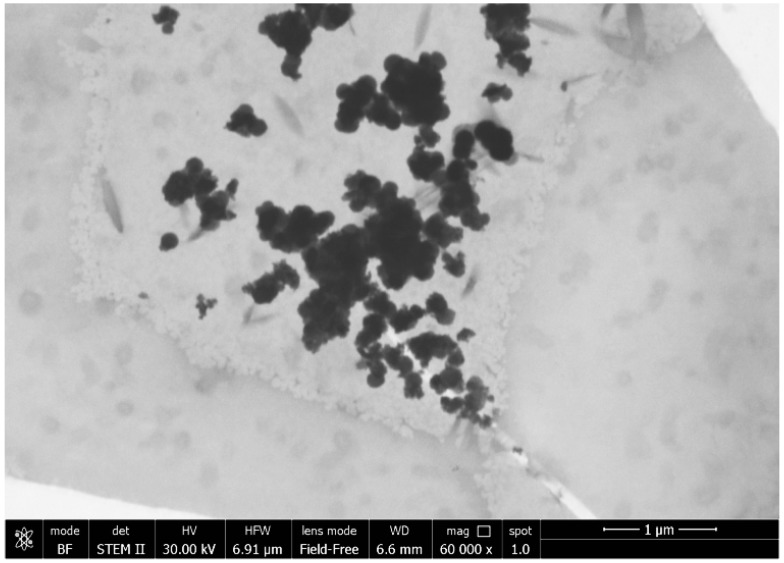
FESEM micrograph of TiO_2_ NPs with C_12_H_28_O_4_Ti:CO(NH_2_)_2_:NH_4_Cl at a ratio of 2:1:0.52, at 50 °C.

The samples with NH_4_Cl (as reported in Table 7) were heterogeneous with a different distribution and the presence of more aggregates (Figure 2); in fact, it is not so easy distinguish the TiO_2_ NPs and their dimensions.

The values of the dimensions calculated from the morphological investigation (obtained from a statistical evaluation of several images) and the diffraction analysis (Debye–Scherrer method) were reported in Table 8; these results indicated a good agreement between the two methods.

### 3.3. Dye Degradation Tests

In this section a qualitative approach regarding the dye degradation is reported and the results are summarized in Table 9a,b.

A similar study was carried out on ceramic tiles. This test was also extended to samples doped with NH_4_Cl and the results are summarized in Table 9b (in this case, there are photos, Figures S10 and S11, reported in the Supplementary Materials).

### 3.4. Bacteriological Tests

Ceramic tiles with a deposition of TiO_2_ NPs (molar ratios C_12_H_28_O_4_Ti:CO(NH_2_)_2_ at a ratio of 2:1, at 50 °C and C_12_H_28_O_4_Ti:CO(NH_2_)_2_:NH_4_Cl at a ratio of 2:1:0.52, at 50 °C) were introduced to the contaminated water.

**Figure 3:**
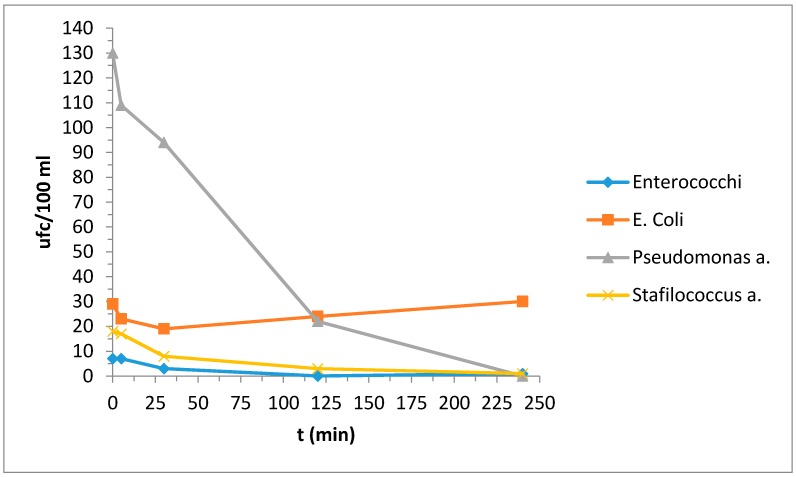
Evaluation of the concentration of the bacterial strains as a function of time.

**Figure 4:**
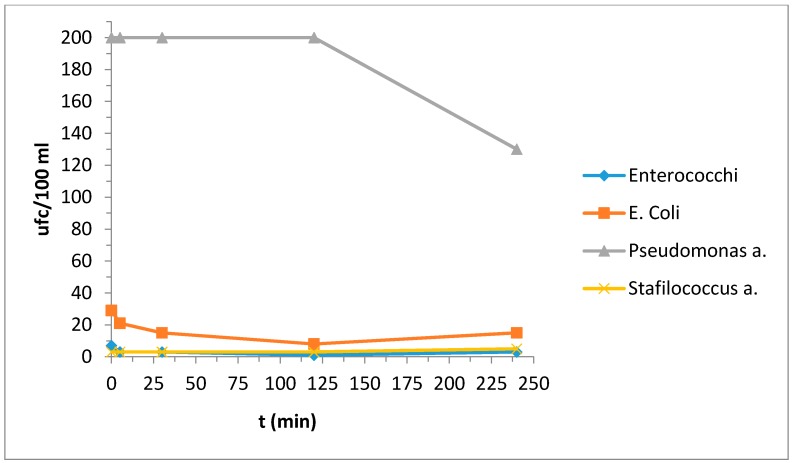
Evaluation of the concentration of the bacterial strains as a function of time.

Table 10a,b reported the bacterial strain values deriving from the contaminated water; for a better interpretation of the behavior with respect to the bacterial strains we also report the same results in Figure 3 and Figure 4.

Photocatalytic properties were evident in the reduction of bacterial strain *Pseudomonas* a.: for the other strains, fluctuations in the results do not allow us to uniquely state that they were actually effectively reduced.

**Figure 5:**
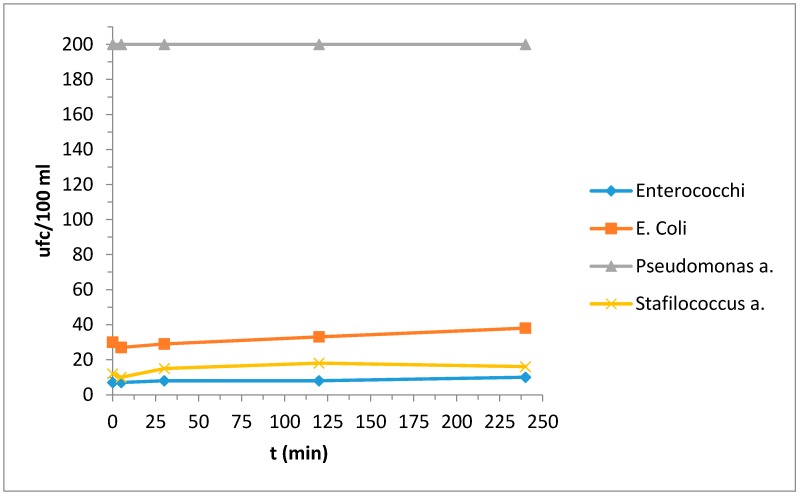
Evaluation of the concentration of the bacterial strains as a function of time.

In this case photocatalytic activity was not evident. We also studied the behavior of a ceramic tile without treatments and the results are reported in Table 11 and Figure 5; in this case the bacterial strain increases with time.

Thus, although there was no evidence of photocatalytic activity in the presence of NH_4_Cl, we can at least say that these samples did not worsen the situation.

These results led us to have clear and complete information on the characteristics of the studied materials.

First of all, the mineralogical analysis of the various samples allowed us to verify the presence of anatase and choose the best synthesis. In fact, we carried out the optimization of Synthesis 2 by varying molar ratios, temperatures and adding NH_4_Cl as a second dopant of nitrogen. In all cases we were able to obtain TiO_2_ in the anatase form.

From a morphogical evaluation we observed the formation of TiO_2_ NPs, almost all spherical and regularly distributed; sometimes the formation of aggregates was observed, which was especially evident with NH_4_Cl. These results confirm those of the mineralogical analysis and the dimension of the TiO_2_ NPs are about 30–40 nm, these dimensions are not considered genotoxic.

The qualitative study of the photocatalytic activity towards the two dyes in the solution and on the ceramic surface indicates that all samples allow complete degradation, except in the case of the sample with NH_4_Cl; the results regarding the ceramic tiles are very important because we will simulate coatings in contact with the water of a public structure.

TiO_2_ NPs with C_12_H_28_O_4_Ti:CO(NH_2_)_2_ in a ratio of 2:1 had photocatalytic properties in reducing the bacterial strain *Psudomonas a*.; samples with NH_4_Cl were not capable of leading to significant variations in the number of bacterial colonies.

Instead, as far as the synthesis with NH_4_Cl as a dopant is concerned, it is evident that the results are worse with respect to those without the dopant, but in term of bacteriological testing the situation is not so tragic. Consequently, this synthesis could be improved with other molar ratios, temperatures, and reaction times.

## 4. Conclusions

The study reported in this manuscript comes from research developed in collaboration with a company that is looking for a synthesis of TiO_2_ NPs that can be easily reproduced in a laboratory at the industrial scale and without dangerous products.

Consequently, the aim of this manuscript was the optimization of old syntheses of TiO_2_ NPs and the verification of their photocatalytic properties for water purification; several syntheses were tested and improved.

Taking into account the obtained results, it can be stated that the synthesis with the best performance of TiO_2_ NPs is that of molar C_12_H_28_O_4_Ti:CO(NH_2_)_2_ in a ratio of 2:1 and at a temperature of 50 °C. 

These are easily reproducible conditions at an industrial level, with low economic impact and without dangerous products. 

## Figures and Tables

**Table 1:** Reagents for Synthesis 1.

Compounds	Amounts
TiCl_4_	5 mL
C_2_H_5_OH	50 mL
H_2_O	200 mL

**Table 2:** Reagents for Synthesis 2.

Compounds	Amounts
C_12_H_28_O_4_Ti	50 mL
H_2_O	200 mL
CO(NH_2_)_2_	1 g

**Table 3:** Reagents for Synthesis 3.

Compounds	Amounts
C_12_H_28_O_4_Ti	3.17 mL
C_3_H_8_O	9.50 mL
CH_3_COOH	10 mL
CH_3_OH	24 mL

**Table 4:** Reagents for Synthesis 4.

Molar Ratios C_12_H_28_O_4_Ti:CO(NH_2_)_2_:NH_4_Cl	Compounds	Amounts
10:1:0	C_12_H_28_O_4_Ti	50 mL
	CO(NH_2_)_2_	1 g
	H_2_O	200 mL
2:1:0	C_12_H_28_O_4_Ti	50 mL
	CO(NH_2_)_2_	5 g
	H_2_O	200 mL
10:1:0.52	C_12_H_28_O_4_Ti	10 mL
	CO(NH_2_)_2_	0.2 g
	NH_4_Cl	0.95 g
	H_2_O	40 mL
2:1:0.52	C_12_H_28_O_4_Ti	10 mL
	CO(NH_2_)_2_	40 mL
	NH_4_Cl	0.2 g
	H_2_O	1.02 g

**Table 5:** Theoretical and experimental values for diffraction analysis.

Anatase			Synthesis 1	Synthesis 2	Synthesis 3
d (Å)	2θ	I (%)	I (%)	I (%)	I (%)
3.52	25.25	100	100	100	–
1.89	47.97	35	14	23	–
2.38	37.80	28	25	32	–

**Table 6:** Theoretical and experimental values for the diffraction analysis.

Anatase			Synthesis 4 Molar Ratio C_12_H_28_O_4_Ti:CO(NH_2_)_2_:NH_4_Cl Temperature					
			10:1:0 50 °C	2:1:0 50 °C	10:1:0 r.t.	2:1:0 r.t.	10:1:0.52 50 °C	2:1:0.52 50 °C
d (Å)	2θ	I (%)	I (%)					
3.52	25.25	100	100	100	100	100	100	100
1.89	47.97	35	32	29	48	19	20	61
2.38	37.80	28	32	30	70	27	19	57

**Table 7:** Morphological characteristic of TiO_2_ NPs.

	Molar Ratio, C_12_H_28_O_4_Ti:CO(NH_2_)_2_:NH_4_Cl Temperature					
	10:1:0 50 °C	2:1:0 50 °C	10:1:0 r.t.	2:1:0 r.t.	10:1:0.52 50 °C	2:1:0.52 50 °C
**Shape**	Irregular	Spherical	Irregular	Spherical	Quite spherical	Quite spherical
**Distribution**	Irregular, aggregates	Regular	Irregular, aggregates	Irregular, aggregates	Irregular, aggregates	Irregular, aggregates

**Table 8:** Dimensions of TiO_2_ NPs calculated with FESEM and XRD analyses.

	Molar Ratios, C_12_H_28_O_4_Ti:CO(NH_2_)_2_:NH_4_Cl Temperature					
	10:1:0 50 °C	2:1:0 50 °C	10:1:0 r.t.	2:1:0 r.t.	10:1:0.52 50 °C	2:1:0.52 50 °C
**XRD (nm)**	27	27	27	27	28	28
**FESEM (nm)**	30	30	40	40	40	30

**Table 9:** (**a**) Qualitative evaluation of the dye degradation for C_12_H_28_O_4_Ti:CO(NH_2_)_2_; (**b**) Qualitative evaluation of the dye degradation for C_12_H_28_O_4_Ti:CO(NH_2_)_2_:NH_4_Cl.

**(a)**		
**Molar Ratios**	**Dyes**	
	**Methyl Orange**	**Bromothymol Blue**
10:1 50 °C	Total degradation after two hours	Total degradation after two hours
2:1 50 °C	Total degradation after four hours	Total degradation after four hours
10:1 r.t.	Total degradation after one hour	Total degradation after one hour
2:1 r.t.	Partial degradation after four hours	Partial degradation after four hours
**(b)**		
**Molar Ratios**	**Dyes**	
	**Methyl Orange**	**Bromothymol Blue**
10:1:0	Low degradation	Total degradation after ½ h
2:1:0	Total degradation after ½ h	Total degradation after ½ h
10:1:0.52	No degradation	No degradation
2:1:0.52	No degradation	Low degradation

**Table 10:** (**a**) Bacterial strains (ufc/100 mL) for C_12_H_28_O_4_Ti:CO(NH_2_)_2_ at a ratio of 2:1, at 50 °C; (**b**) Bacterial strains (ufc/100 mL) for C_12_H_28_O_4_Ti:CO(NH_2_)_2_:NH_4_Cl at a ratio of 2:1:0.52, at 50 °C.

**(a)**					
**Bacterial Strains**	**t (min)**				
	0	5	30	120	240
*Enterococchi*	7	7	3	0	1
*E. coli*	29	23	19	24	30
*Pseudomonas a.*	130	109	94	22	0
*Stafilococcus a.*	18	17	8	3	1
**(b)**					
**Bacterial Strains**	**t (min)**				
	0	5	30	120	240
*Enterococchi*	7	3	3	1	3
*Escherichia coli*	29	21	15	8	15
*Pseudomonas a.*	200	200	200	200	130
*Stafilococcus a.*	3	3	3	3	5

**Table 11:** Bacterial strains (ufc/100 mL).

Bacterial Colonies	t (min)				
	0	5	30	120	240
*Enterococchi*	7	7	8	8	10
*Escherichia coli*	30	27	29	33	38
*Pseudomonas aeruginosa*	200	200	200	200	200
*Stafilococcus aureus*	12	10	15	18	16

## Supplementary Materials

The following are available online at www.mdpi.com/1996-1944/10/10/1208/s1, Figure S1: XRPD pattern of sample of Synthesis 1; Figure S2: XRPD pattern of sample of Synthesis 2; Figure S3: XRPD pattern of sample of Synthesis 3; Figure S4: XRPD pattern of sample with molar ratio C_12_H_28_O_4_Ti:CO(NH_2_)_2_:NH_4_Cl 10:1: 0, 50 °C; Figure S5: XRPD pattern of sample with molar ratio C_12_H_28_O_4_Ti:CO(NH_2_)_2_:NH_4_Cl 2:1: 0, 50 °C; Figure S6: XRPD pattern of sample with molar ratio C_12_H_28_O_4_Ti:CO(NH_2_)_2_:NH_4_Cl 10:1: 0, r.t.; Figure S7: XRPD pattern of sample with molar ratio C_12_H_28_O_4_Ti:CO(NH_2_)_2_:NH_4_Cl 2:1: 0, r.t.; Figure S8: XRPD pattern of sample with molar ratio C_12_H_28_O_4_Ti:CO(NH_2_)_2_:NH_4_Cl 10:1: 0.52, 50 °C; Figure S9: XRPD pattern of sample with molar ratio C_12_H_28_O_4_Ti:CO(NH_2_)_2_:NH_4_Cl 2:1: 0.52, 50 °C; Figure S10: Ceramic titles with molar ratio C_12_H_28_O_4_Ti:CO(NH_2_)_2_ 10:1 (left part of image) and 2:1 (right part of image), 50 °C, before (a) and after (b) ½ hour of sunlight exposure; Figure S11: Ceramic titles with molar ratio C_12_H_28_O_4_Ti:CO(NH_2_)_2_:NH_4_Cl 10:1:0.52 (left part of image) and 2:1: 0.52 (right part of image), 50 °C, before (a) and after (b) ½ hour of sunlight exposure;
