# Supplementary material for: Synthesis and Characterization of TiO2 Nanoparticles for the Reduction of Water Pollutants
Source: Materials (Basel). 2017 Oct 20;10(10):1208. doi: 10.3390/ma10101208 (PMC5667014; doi:10.3390/ma10101208)
Supplement: Supplementary file 1 [file materials-10-01208-s001.pdf]

materials

*Article**

Synthesis and Characterization of TiO_2 Nanoparticles for the Reduction of Water Pollutants

Gigliola Lusvardi [1,*], Corrado Barani [2], Federica Giubertoni [2] and Giulia Paganelli [1]

[1] Department of Chemistry and Geological Sciences, University of Modena and Reggio Emilia, Via G. Campi 103, 41125 Modena, Italy; giulipez91@gmail.com

[2] Barchemicals, Via S. Allende 14, 41051 Castelnuovo Rangone (MO), Italy; barani.corrado@barchemicals.it (C.B.); giubertoni.federica@barchemicals.it (F.G.)

* Correspondence: gigliola.lusvardi@unimore.it, Tel.: +39-059-205-8549

Received: 30 August 2017; Accepted: 18 October 2017; Published: date

Supplementary Materials

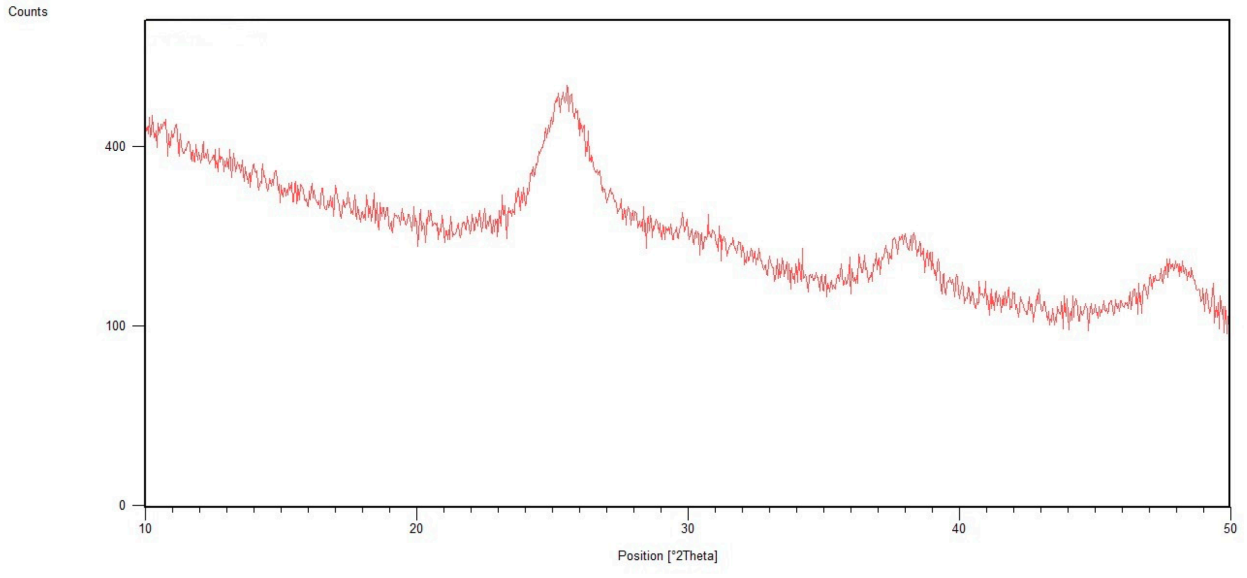

Figure S1. XRPD pattern of sample of Synthesis 1.

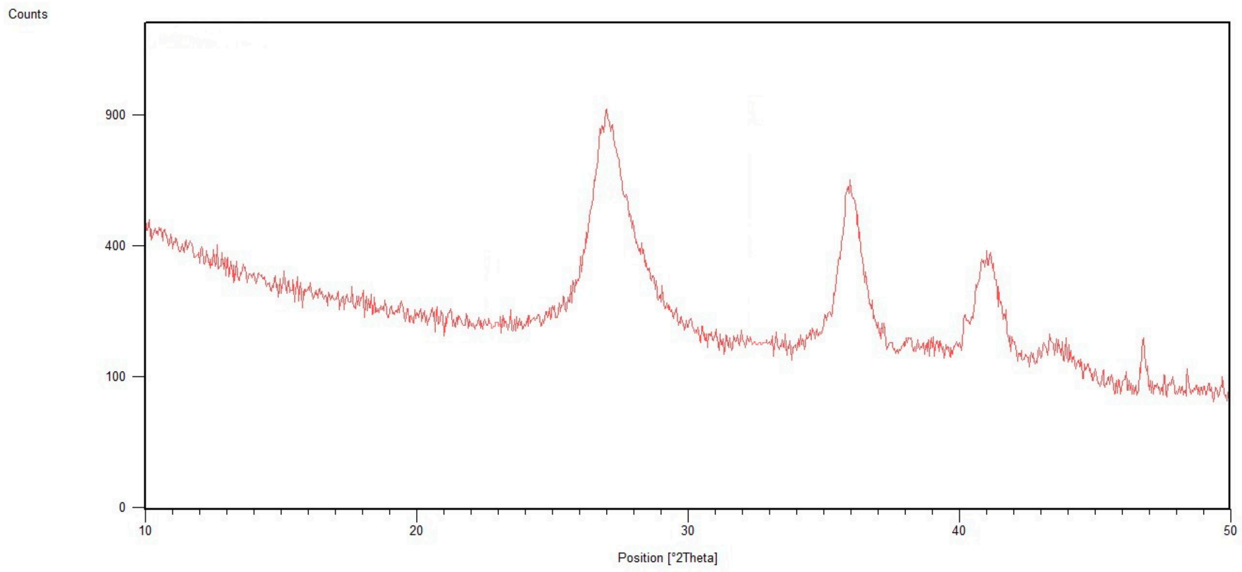

Figure S2. XRPD pattern of sample of Synthesis 2.

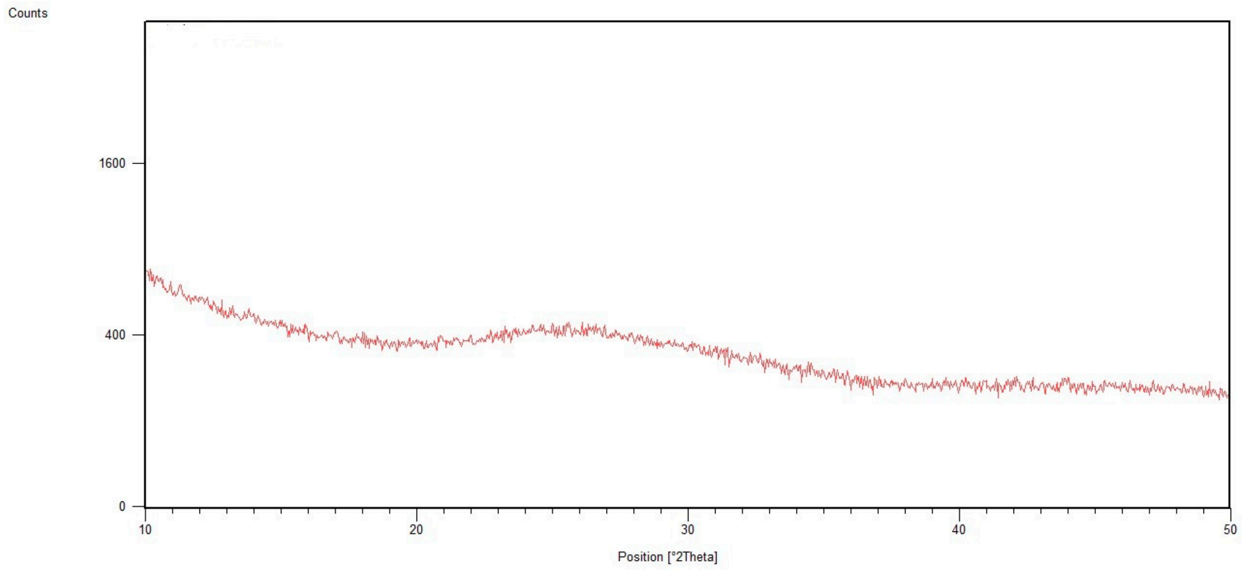

Figure S3. XRPD pattern of sample of Synthesis 3.

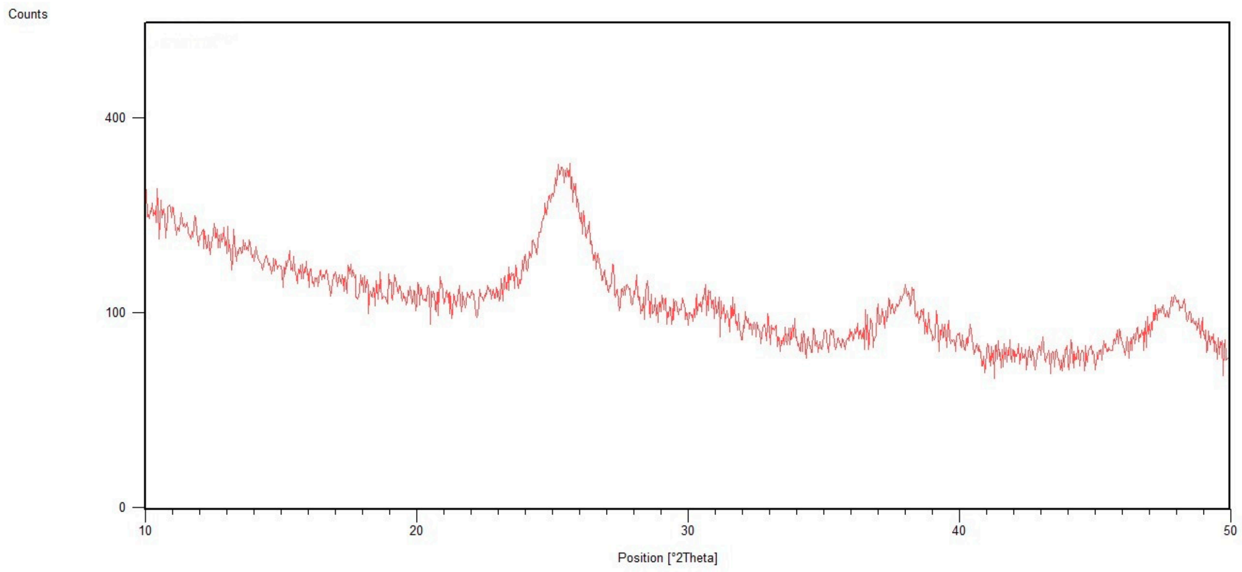

Figure S4. XRPD pattern of sample with molar ratio $C_{12}H_{28}O_4Ti$:$CO(NH_2)_2$:NH_4Cl 10:1: 0, 50 °C.

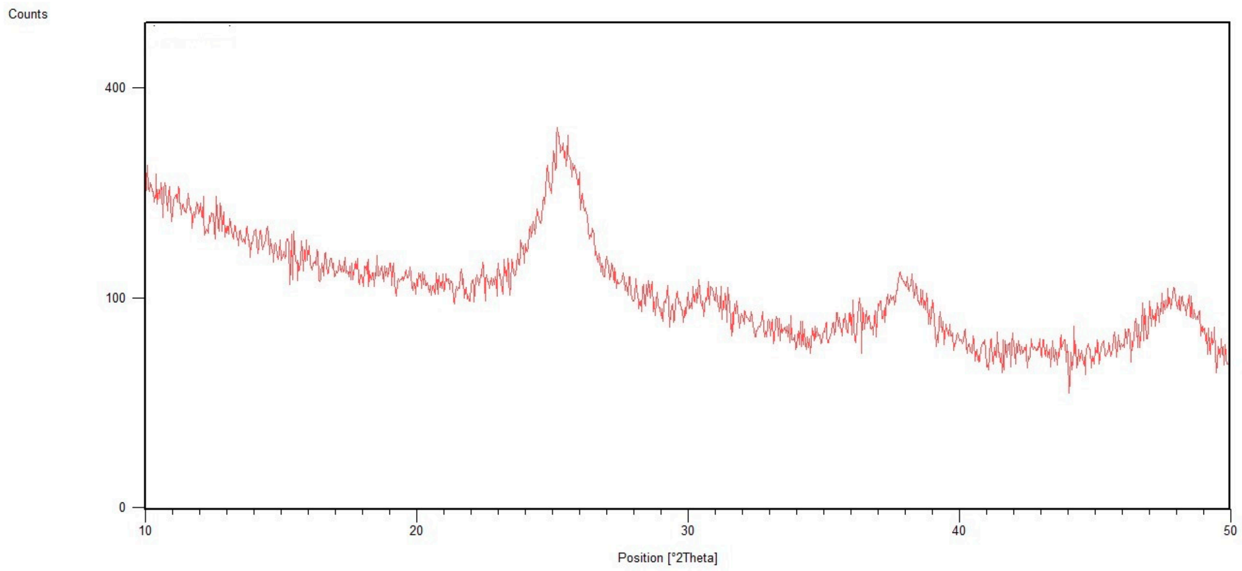

Figure S5. XRPD pattern of sample with molar ratio $C_{12}H_{28}O_4Ti$:$CO(NH_2)_2$:NH_4Cl 2:1: 0, 50 °C.

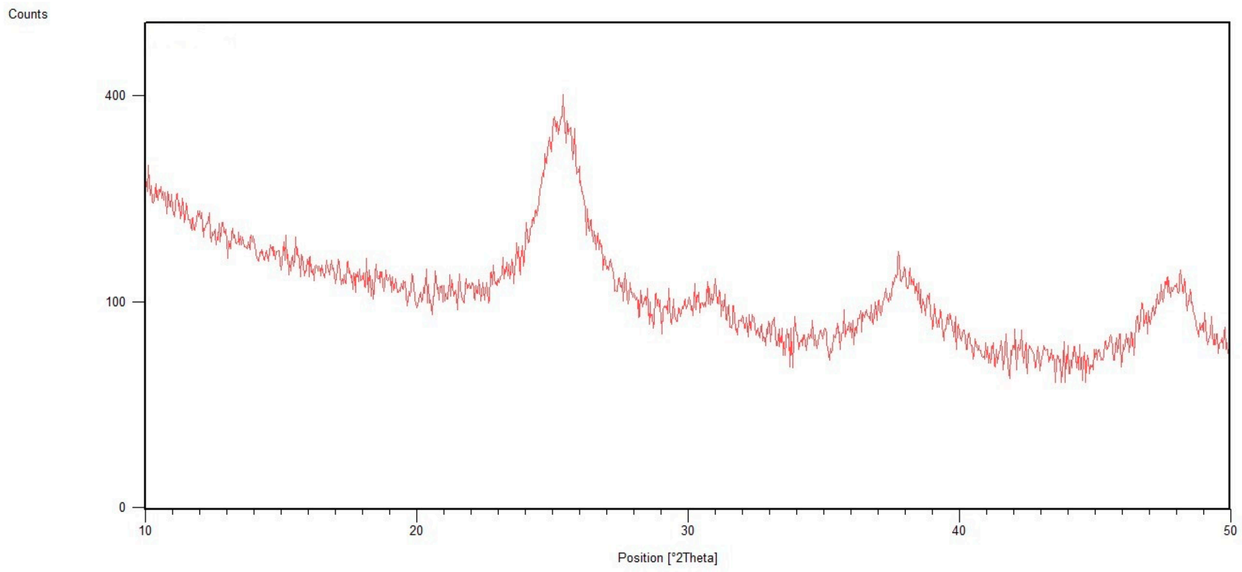

Figure S6. XRPD pattern of sample with molar ratio $C_{12}H_{28}O_4Ti$:$CO(NH_2)_2$:NH_4Cl 10:1: 0, r.t.

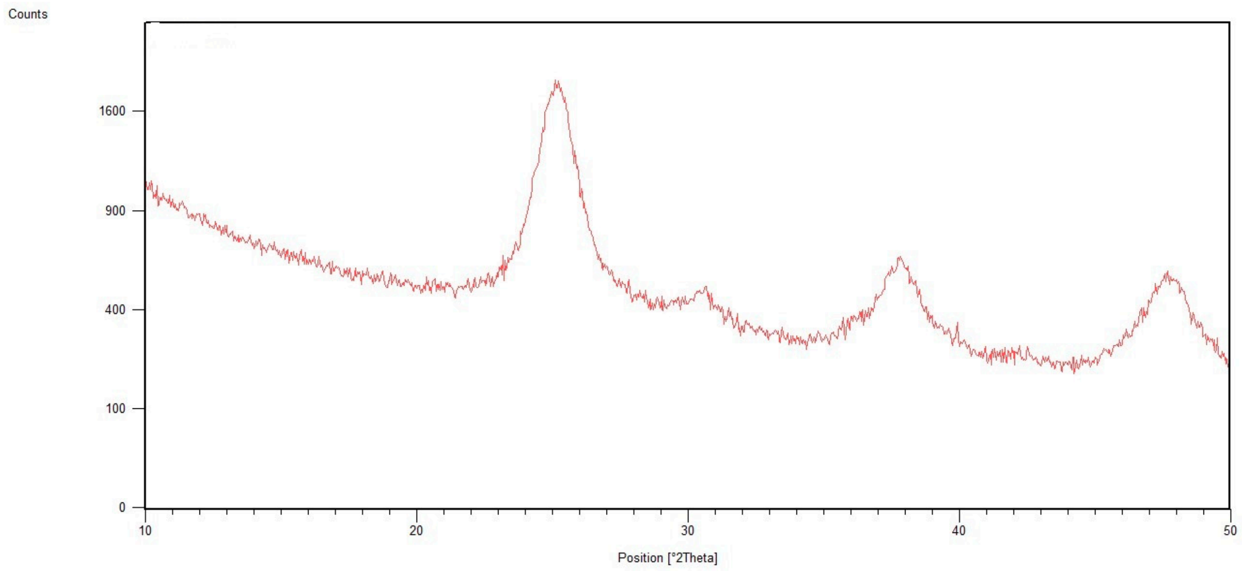

Figure S7. XRPD pattern of sample with molar ratio $C_{12}H_{28}O_4Ti$:$CO(NH_2)_2$:NH_4Cl 2:1: 0, r.t.

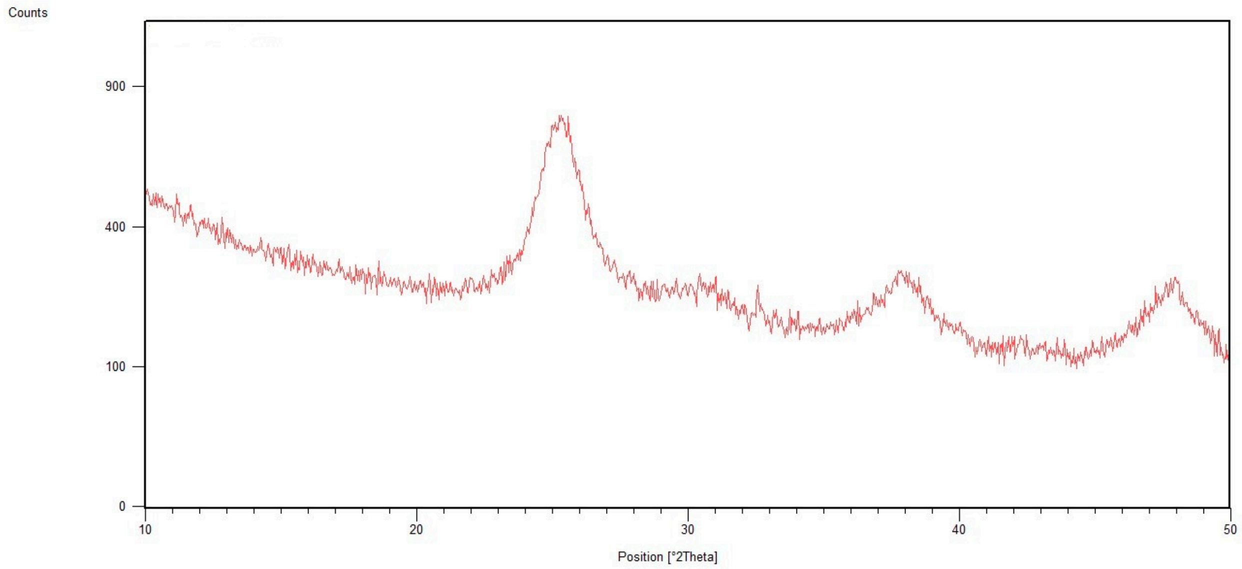

Figure S8. XRPD pattern of sample with molar ratio $C_{12}H_{28}O_4Ti$:$CO(NH_2)_2$:NH_4Cl 10:1: 0.52, 50 °C.

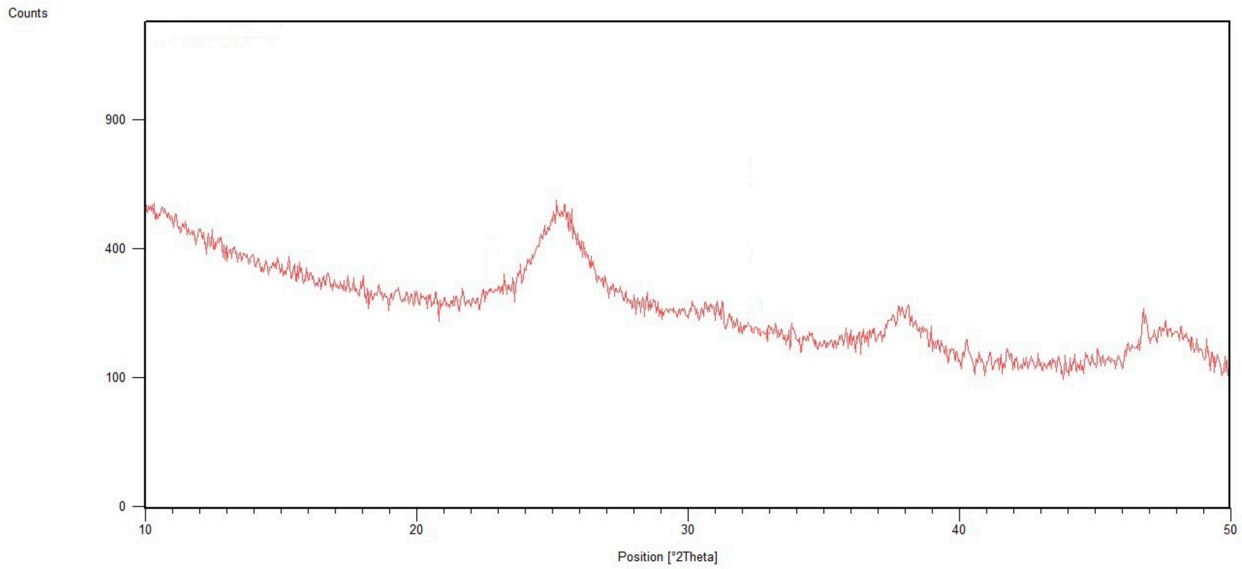

Figure S9. XRPD pattern of sample with molar ratio $C_{12}H_{28}O_4Ti$:$CO(NH_2)_2$:NH_4Cl 2:1: 0.52, 50 °C.

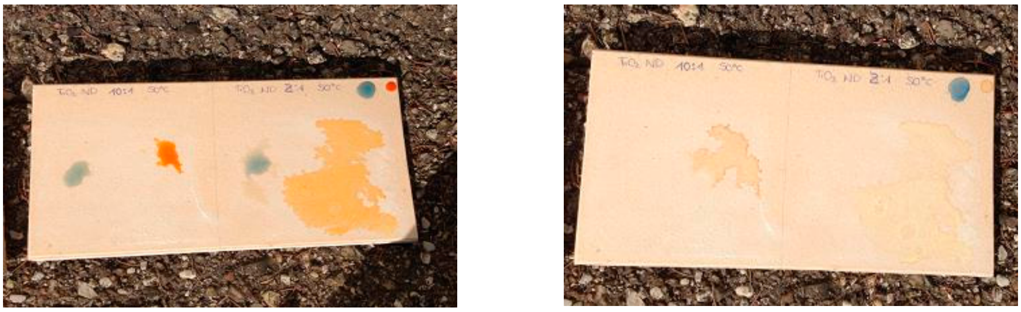

(a) (b)

Figure S10. Ceramic titles with molar ratio $C_{12}H_{28}O_4Ti$:$CO(NH_2)_2$ 10:1 (left part of image) and 2:1 (right part of image), 50 °C, before (a) and after (b) ½ hour of sunlight exposure.

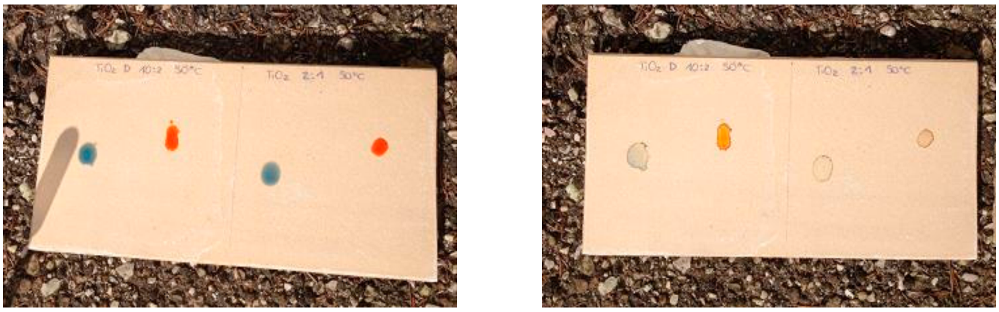

(a) (b)

Figure S11. Ceramic titles with molar ratio $C_{12}H_{28}O_4Ti$:$CO(NH_2)_2$:NH_4Cl 10:1:0.52 (left part of image) and 2:1: 0.52 (right part of image), 50 °C, before (a) and after (b) ½ hour of sunlight exposure.

© 2017 by the authors. Submitted for possible open access publication under the terms and conditions of the Creative Commons Attribution (CC BY) license (http://creativecommons.org/licenses/by/4.0/).
